# Clinical efficacy of early postoperative intensity-modulated radiotherapy combined with Temozolomide chemotherapy in the treatment of patients with malignant glioma

**DOI:** 10.12669/pjms.38.6.5244

**Published:** 2022

**Authors:** Hongyang Zhou, Huijie Wu, Meng Li, Chao Dong, Tongyou Sun

**Affiliations:** 1 Hongyang Zhou, Anesthesiology Department, Chengde Central Hospital, Chengde 067000, Hebei, China; 2 Huijie Wu, Chemo radiotherapy Center, Chengde Central Hospital, Chengde 067000, Hebei, China; 3 Meng Li, Chemo radiotherapy Center, Chengde Central Hospital, Chengde 067000, Hebei, China; 4 Chao Dong, Reproductive Medicine Center, Chengde Central Hospital, Chengde 067000, Hebei, China; 5 Tongyou Sun, Chemo radiotherapy Center, Chengde Central Hospital, Chengde 067000, Hebei, China

**Keywords:** Early postoperative period, Intensity-modulated radiotherapy, Temozolomide, Malignant glioma, Clinical efficacy

## Abstract

**Objectives:**

To evaluate the clinical efficacy of early postoperative intensity-modulated radiotherapy (IMRT) combined with temozolomide chemotherapy in the treatment of patients with malignant glioma.

**Methods:**

In this retrospective cohort study 80 patients with glioma surgery admitted to Chengde Central Hospital from January 2019 to January 2021 were selected and divided into two groups according to postoperative treatment: the experimental group and the control group, with 40 cases in each group. Patients in the experimental group received IMRT combined with temozolomide chemotherapy postoperatively, while those in the control group received IMRT alone. The clinical effects of patients were analyzed before treatment and three months after treatment, and the incidence of adverse reactions such as bone marrow suppression, gastrointestinal reactions, fever, and liver dysfunction were analyzed in the two groups within one month after treatment. Before treatment and two months after treatment, MMSE scale, QOL scale and KPS were used to compare the cognitive function and health status of the patients. All patients were followed up for one year after treatment, and the difference of disease progression-free survival and overall survival rate between the two groups was analyzed.

**Results:**

The effective rate of the experimental group was 70% after treatment, while that of the control group was 43.3%, with a statistically significant difference (P=0.04). The incidence of adverse reactions was 50% in the experimental group and 40% in the control group, with no statistically significant difference between the two groups (P=0.25). After treatment, MMSE score, QOL score and KPS score of the experimental group were significantly improved compared with those of the control group, with statistically significant differences between the two groups (MMSE score, QOL, P=0.00; KPS, P=0.01). Moreover, the two groups of patients were followed up for one year after treatment. The disease progression-free survival rate of the experimental group was 70% and that of the control group was 47.5%, with a statistically significant difference (P=0.04), and the overall survival rate of the experimental group was significantly higher than that of the control group after treatment, with a statistically significant difference (P=0.03).

**Conclusion:**

Early postoperative IMRT combined with temozolomide chemotherapy is an effective treatment regimen for patients with malignant glioma, boasting a variety of advantages such as high efficiency, cognitive function, favorable recovery of health status, significantly improved progression-free survival rate and overall survival rate, and no significant increase in adverse reactions.

## INTRODUCTION

Glioma originates from glial stem cells or progenitor cells and is the most common brain malignant tumor clinically,^1^ accounting for approximately 30% of all central system tumors and 80% of malignant brain tumors. It is characterized by rapid progression, 5-year survival rate less than 10% and poor prognosis.^2^ The current standard treatment for glioma is surgical treatment, followed by postoperative radiotherapy and chemotherapy.^3^ It is considered by Molinaro et al.^4^ that the biological characteristics of gliomas are swelling and infiltrating growth, leading to a high recurrence rate, faster growth rate of recurrent glioma, more aggressive and poorer prognosis. In view of this, no standard treatment regimen for glioma has been proposed. Targeted therapy has little effect on gliomas. All therapies with significant survival benefits for gliomas, including radiation and chemotherapy, were investigated in phase III trials.^5^ Therefore, most researchers^6^ currently tend to support maximum surgical resection of gliomas on the basis of nerve function preservation, followed by postoperative radiotherapy or chemotherapy. Since the pathogenesis and mechanism of glioma are multifactorial, clinical treatment decisions should also be based on multi-factor considerations.^7^ Both IMRT and temozolomide chemotherapy are approaches to the treatment of tumor diseases, but the two differ in the mechanism of action. In this study, IMRT combined with temozolomide chemotherapy was used for the treatment of patients with malignant glioma after surgery, and a certain therapeutic effect was achieved.

## METHODS

This was a retrospective cohort study. Eighty patients with glioma surgery admitted to Chengde Central Hospital from January 2019 to January 2021 were selected and divided into two groups according to postoperative treatment: the experimental group and the control group, with 40 cases in each group. The sample size required for each group was calculated by the formula 
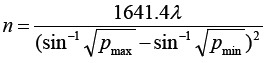
. Patients in the experimental group received IMRT combined with temozolomide chemotherapy postoperatively, while those in the control group received IMRT alone. Among them, there were 21 males and 19 females in the experimental group, aged 44-75 years with an average of 59.33±9.96 years, and 23 males and 17 females in the control group, aged 43-73 years with an average of 59.83±9.52 years. No significant difference can be seen in the comparison of general data between the two groups, which was comparable between the two groups (Table-I).

**Table I:** Comparative analysis of general data between the experimental group and the control group (*χ̅*±S) n=40.

Indicators	Experimental group	Control group	t/χ^2^	P
Age (years old)	59.33±9.96	59.83±9.52	0.23	0.82
Male (%)	21	23	0.20	0.65
Operational				
Total excision	31	27	1.00	0.32
Partial excision	9	13		
Tumor diameter (cm)				
≥ 6cm	11	14	0.52	0.47
< 6cm	29	26		
Whether the center line is crossed				
No	33	31	0.31	0.58
Yes	7	9		
Pathologic result				
Stellate cells	27	29	0.24	0.63
No stellate cells	13	11		

P>0.05.

### Ethical Approval

The study was approved by the Institutional Ethics Committee of Chengde Central Hospital on October 26, 2021 (No.202109A182), and written informed consent was obtained from all participants

### Inclusion criteria:

• All patients who underwent surgical treatment and whose pathological findings were diagnosed as glioma;^1^
• Patients younger than 75 years old;
• Patients with an expected survival period of more than 6 months;
• Patients with complete clinical data
• Patients whose tumor size can be accurately measured by preoperative CT or MRI and other imaging data;
• Patients whose family members are willing and able to complete the study and have good treatment compliance;
• Patients who have signed an informed consent form.

### Exclusion criteria:

• Patients with poor physique and unstable vital signs who cannot tolerate treatment;
• Patients with other systemic malignancies;
• Patients with serious underlying diseases and contraindications to surgery, radiotherapy and chemotherapy;
• Patients with mental and nervous system abnormalities or unable to complete the study due to other reasons.

### Surgical methods

All patients underwent surgery under conventional intraoperative imaging navigation or imaging combined with neuro-electrophysiological detection, and total or partial tumor resection was performed according to the specific intraoperative conditions. In terms of the degree of surgical resection, total resection means no postoperative residual, while partial resection means postoperative residual.

Patients in the control group received IMRT alone, with primary radiotherapy beginning immediately after complete healing of the incision. Patients were placed in supine position with their heads fixed. The labeled target area was determined according to the CT and MRI results before and after surgery. The Varian 2300C linear accelerator was used for 6 mV-X-ray treatment. Method of radiographic target division: MRI T2WI/FLAIR sequence was fused with localized CT, and T2WI/FLAIR hypersignal area was used as tumor target (GTV). The clinical target area (CTV) was expanded by 1-2 cm from GTV, and the planned target area (PTV) was obtained by expanding 0.5cm from CTV. The single dose of irradiation was 1.8-2.0 Gy, and the total dose was 45-54 Gy, once per day.

Patients in the experimental group were treated with 150mg/m^2^ of temozolomide on the basis of IMRT, once a day, 5 times/week, 28d as a cycle, and then changed to 200mg/m2 dose treatment for a total of three cycles.

### Observation Indicators:

### Evaluation of clinical efficacy

Patients were re-examined three months postoperatively, and their clinical efficacy was divided according to MRI or CT: Complete remission (CR): the tumor disappears completely, and no new lesions appear for four weeks; Partial remission (PR): tumor shrinkage ≥ 50%, and no new lesions appear after four weeks; Stable disease (SD): tumor shrinkage < 50%, and no new lesions appear after four weeks; Progressive disease (PD): tumor grows larger or new lesions appear. Total effective = complete remission + partial remission/100. Evaluation of adverse drug reactions: Adverse drug reactions, including bone marrow suppression, gastrointestinal reactions, fever, liver dysfunction and other adverse reactions, were recorded in the two groups within one month after medication.

### Evaluation of cognitive function and health status

The MMSE scale^8^ was used to evaluate the cognitive function of patients before treatment and 2 months after treatment, and the higher the score, the better the cognitive function of patients. The QOL scale^9^ was used to evaluate the quality of life of patients, and the higher the score, the better the quality of life of patients. KPS was used to evaluate the health status of patients, and the higher the score, the better the health status of patients.

### Postoperative follow-up

All patients were followed up for one year after the end of treatment, and the prognosis of the patients was analyzed, including disease-free survival rate and overall survival rate.

### Statistical Analysis

All the data were statistically analyzed by SPSS 20.0 software, and the measurement data were expressed as (X̅± s). Two independent sample t-test was used for inter-group data analysis, paired t test was used for intra-group data analysis, and c^2^ was adopted for rate comparison. P<0.05 indicates a statistically significant difference.

## RESULTS

Analysis of the effective rate of the two groups showed that the effective rate of the experimental group was 70%, which was significantly higher than that of the control group (43.3%), with a statistically significant difference (P=0.02, Table-II.

**Table II:** Comparative analysis of the clinical efficacy of the two groups (*χ̅*±S) n=40.

Group	CR	PR	SD	PD	Total effective rate
Experimental group	16	14	6	4	30(70%)
Control group	12	9	11	8	21(43.3%)
c^2^					4.38
P					0.04

p<0.05.

The comparative analysis of the incidence of adverse drug reactions between the two groups after treatment showed that the incidence of adverse reactions in the experimental group was 50%, which was higher than that in the control group (40%), with no statistical significance (P=0.25) Table-III.

**Table III:** Comparative analysis of adverse drug reactions between the two groups after treatment (*χ̅*±S) n=40.

Group	Bone marrow suppression	Gastrointestinal reaction	Fever	Liver function damage	Incidence
Experimental group	4	3	5	5	17(50%)
Control group	3	4	1	4	12(40%)
χ^2^					1.35
P					0.25

p<0.05.

No statistically significant difference can be seen in the comparison of MMSE score, QOL score and KPS score between the experimental group and the control group before treatment (P>0.05). MMSE score, QOL score and KPS score of the experimental group improved significantly compared with those of the control group after treatment, with a statistically significant difference between the two groups (MMSE score, QOL, P=0.00; KPS, P=0.01, Table-IV

**Table IV:** Comparative analysis of cognitive function and health status of the two groups before and after treatment (*x̅*±S) n=40.

Scoring indicators		Experimental group	Control group	t	p
MMSE score	Before treatment	16.58±3.73	16.47±3.19	0.14	0.89
	After treatment*	24.76±3.05	21.78±2.60	4.70	0.00
QOL score	Before treatment	2.98±0.37	2.86±0.28	1.64	0.11
	After treatment*	3.87±0.74	3.35±0.81	2.99	0.00
KPS score	Before treatment	53.46±7.85	53.39±6.95	0.04	0.96
	After treatment*	74.31±9.41	68.37±7.59	3.11	0.01

* p<0.05.

**Fig.1:**
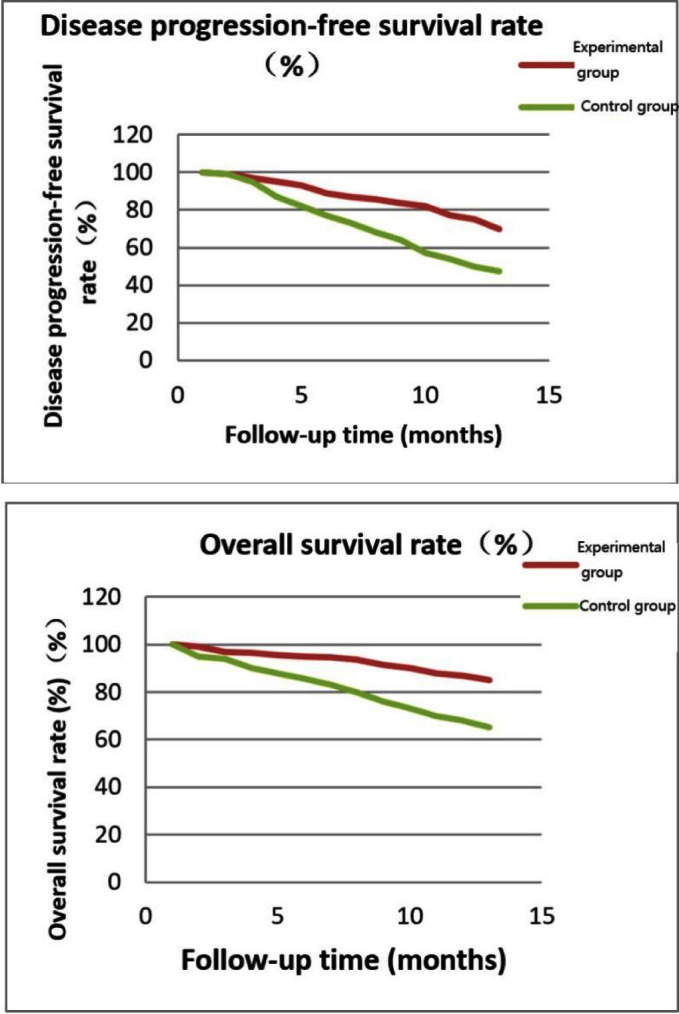
Comparative analysis of postoperative follow-up between the two groups.

Patients in the two groups were followed up for one year after treatment. The disease progression-free survival rate was 70% in the experimental group and 47.5% in the control group, with a statistically significant difference (P=0.04). The overall survival rate of the experimental group was significantly higher than that of the control group after treatment, with a statistically significant difference (P=0.03) Fig.1

## DISCUSSION

Glioma, as a clinically common primary intracranial malignant tumor, often invades people over 40 years old. Its important feature is local vascular dysplasia^10^, and is characterized by rapid growth, strong invasiveness and high degree of malignancy in high-grade cases, which seriously affects the quality of life of patients and has a high mortality.^2^ Malignant glioma is usually accompanied by a very poor efficacy and prognosis due to the existence of blood-brain barrier and various drug resistance mechanisms of this tumor, and conventional treatment regimens are often ineffective in treating this tumor.^11^

Surgical treatment is currently the preferred method for clinical treatment of gliomas.^12^ Surgical resection of tumors boasts of reducing tumor burden, clarifying pathological diagnosis and guiding further treatment, which plays an important role in the treatment of glioma, and the degree of surgical resection affects the prognosis. It was proposed in the study of Smith et al.^13^ that if the surgical resection volume exceeds 90% of the tumor, the 5-year OS is about 97%, and if it is less than 90%, the 5-year OS will drop to 76%. It was further proposed in the study of Kavouridis et al. 14 that total tumor resection could delay the time of tumor progression. However, the total resection rate of pure surgery is still low, and patients still have a high risk of recurrence after surgery.^15^ Therefore, for patients with glioma surgery, radiotherapy and chemotherapy are often used as adjuvant treatments after surgery to further improve the effect of disease control and reduce disease recurrence.^16^

IMRT is a new radiotherapy concept proposed in recent years^17^, which belongs to three-dimensional conformal radiotherapy. Its intensity can be adjusted according to the specific anatomical structure of the target area, so as to promote the uniformity of the entire target area and reduce radiation damage while ensuring the effect of radiotherapy. A retrospective study involving 220 patients by Thibouw et al.^18^ believed that IMRT has improved the consistency of the target in patients with glioblastoma and significantly reduced neurotoxicity via a comparative analysis of 3D-CRT and IMRT-treated glioblastoma. It was believed by Eekers et al.^19^ that IMPT can significantly reduce radiation dose in most patients compared with conventional brain radiation therapy, resulting in a significant reduction in neurocognitive decline and an improvement in quality of life. It was confirmed in this study that the MMSE score, QOL score and KPS score of the experimental group were significantly improved compared with those of the control group after treatment, with statistically significant differences between the two groups (MMSE score, QOL, P=0.00; KPS, P=0.01), which was similar to the results of previous studies. However, Eekers et al.^20^ concluded that patients receiving early radiotherapy had better seizure control within one year than those receiving delayed radiotherapy, and showed differences in memory, executive function, cognitive function, or quality of life compared with patients receiving late radiation therapy.

Multiple drug resistance mechanisms and pathogenic mechanisms of tumors indicate that a single treatment is less effective than an overall treatment regimen.^21^ Temozolomide is an imidazolazine antitumor drug that inhibits the activity of guanine in tumor DNA to inhibit tumor replication. It can provide survival benefit for patients with glioblastoma^22^ and is the first-line treatment for glioblastoma.^23^,^24^ It was suggested in the study of Mackay et al.^25^ that combined radiotherapy may improve glioma cell promoters and improve survival in patients with methylated glioblastoma compared with standard temozolomide therapy alone. In our study, the two groups of patients were followed up for one year after treatment. The disease progression-free survival rate was 70% in the experimental group and 47.5% in the control group, with a statistically significant difference (P=0.04). The overall survival rate of the experimental group was significantly higher than that of the control group after treatment, with a statistically significant difference (P=0.03), which was similar to the results of Mackay et al. It was also confirmed in this study that the effective rate of the experimental group was 70% after treatment, which was significantly higher than that of the control group (43.3%), with a statistically significant difference (P=0.04). It was believed by Weller et al.^26^ that compared with temozolomide alone, the survival benefit after combined radiotherapy was significantly different, and the cognitive function and health status of patients were also significantly improved. This can provide support for the results of our study.

### Limitations of this study

Nevertheless, shortcomings can still be seen in this study: Fewer samples and short follow-up time make it impossible to evaluate the long-term prognosis of patients with malignant glioma treated with IMRT combined with temozolomide chemotherapy. Moreover, more immunotherapy and targeted therapy drugs have been applied in clinical practice with the in-depth study on tumor immunotherapy and targeted therapy, but the treatment regimen described in this paper has not yet been included in these treatment regimens. In response to this, proactive countermeasures will be taken to increase the sample size and further prolong the follow-up time. New cancer treatments and related drugs will be added for comparative analysis with this study, so as to make a more objective evaluation of the efficacy of this treatment regimen.

## CONCLUSION

Early postoperative IMRT combined with temozolomide chemotherapy is an effective treatment regimen for patients with malignant glioma, boasting a variety of advantages such as high efficiency, cognitive function, favorable recovery of health status, significantly improved progression-free survival rate and overall survival rate, and no significant increase in adverse reactions.

### Authors’ Contributions:

**HZ** & **HW:** designed this study and prepared this manuscript, are responsible and accountable for the accuracy and integrity of the work. **ML** & **CD:** Collected and analyzed clinical data. **TS:** Significantly revised this manuscript.
